# The Prosigna 50-gene profile and responsiveness to adjuvant anthracycline-based chemotherapy in high-risk breast cancer patients

**DOI:** 10.1038/s41523-020-0148-0

**Published:** 2020-02-26

**Authors:** Maj-Britt Jensen, Anne-Vibeke Lænkholm, Eva Balslev, Wesley Buckingham, Sean Ferree, Vesna Glavicic, Jeanette Dupont Jensen, Ann Søegaard Knoop, Henning T. Mouridsen, Dorte Nielsen, Torsten O. Nielsen, Bent Ejlertsen

**Affiliations:** 1 Danish Breast Cancer Cooperative Group, Rigshospitalet, Copenhagen University Hospital, Copenhagen, Denmark; 2 Department of Surgical Pathology, Zealand University Hospital, Slagelse, Denmark; 3 Department of Pathology, Herlev and Gentofte Hospital, University of Copenhagen, Herlev, Denmark; 4 NanoString Technologies Inc, Seattle, WA USA; 5 Department of Oncology, Zealand University Hospital, Naestved, Denmark; 6 Department of Oncology, Odense University Hospital, Odense, Denmark; 7 Department of Oncology, Rigshospitalet, Copenhagen University Hospital, Copenhagen, Denmark; 8 Department of Oncology, Herlev and Gentofte Hospital, University of Copenhagen, Herlev, Denmark; 9 Department of Pathology and Laboratory Medicine, University of British Columbia, Vancouver, BC Canada; 10 Danish Breast Cancer Cooperative Group, Department of Oncology, Rigshospitalet, Copenhagen University Hospital, Copenhagen, Denmark

**Keywords:** Predictive markers, Randomized controlled trials

## Abstract

The DBCG89D trial randomized high-risk early breast cancer patients to adjuvant CMF (cyclophosphamide, methotrexate and fluorouracil) or CEF (cyclophosphamide, epirubicin and fluorouracil). Prosigna assays were performed by researchers with no access to clinical data. Time to distant recurrence (DR) was the primary endpoint, time to recurrence (TR) and overall survival (OS) secondary. Among the 980 Danish patients enrolled, Prosigna results were obtained in 686. Continuous ROR score was associated with DR for CMF (adjusted hazard ratio (HR) 1.20, 95% CI 1.09–1.33), and for CEF (HR 1.04, 95% CI 0.92–1.18), *P*_interaction_ = 0.06. DR was significantly longer in CEF compared to CMF treated patients with Her2-enriched tumors (HR 0.58, 95% CI 0.38–0.86), but not in patients with luminal tumors. Heterogeneity of treatment effect was significant for TR and OS. In this prospective-retrospective analysis, patients with Her2-enriched breast cancer derived substantial benefit from anthracycline chemotherapy whereas anthracyclines are not an essential component of chemotherapy for patients with luminal subtypes. The benefit of CEF vs. CMF correlated with increasing ROR Score.

## Introduction

Adjuvant chemotherapy has for decades been a central treatment component for patients with early-stage breast cancer and moderate or high risk of recurrence.^1,2^ Proven clinical utility of the prognostic ability by different tumor transcriptome analyses has now facilitated a decrease in the use of adjuvant chemotherapy among patients with estrogen receptor (ER) positive breast cancers.^3–5^ No benefit was observed in the Trial Assigning IndividuaLized Options for Treatment Rx (TAILORx) trial from adding chemotherapy to endocrine therapy among women with node-negative, ER positive breast cancer and a recurrence score of 11 to 25.^5^ The 70-gene MammaPrint assay was used in the Microarray In Node-negative Disease may Avoid ChemoTherapy (MINDACT) trial and neither patients with low clinical and high genomic risk nor patients with high clinical and low genomic risk obtained substantial benefit from adjuvant chemotherapy.^6^

Most patients with sufficient clinical and genomic risk to warrant adjuvant chemotherapy will receive alkylator-, anthracycline-, and taxane-based chemotherapy but genomic assays have identified patients who are unlikely to benefit from cyclophosphamide-based regimens. Lack of benefit from adjuvant CMF or cyclophosphamide was predicted by the Prosigna Risk Of Recurrence (ROR) score and by intrinsic subtypes in a recent retrospective analysis of the Danish Breast Cancer Group (DBCG) 77B trial that randomized premenopausal high-risk patients with early breast cancer to adjuvant chemotherapy vs. no chemotherapy.^7^ A Basal-like subtype was associated with an 86% reduction in disease-free survival (DFS) event while no benefit was obtained by patients with an HER2-enriched subtype. Patients with a Luminal B subtype had a significant 52% reduction in DFS events while no significant benefit (39% improved DFS) was obtained by patients with a Luminal A subtype.^7^

Greater benefits were on average achieved by anthracycline containing regimens as compared to CMF-based adjuvant chemotherapy.^1,2^ Anthracyclines are cardiotoxic and leukemogenic, but in the Early Breast Cancer Trialists’ Collaborative Group (EBCTCG) meta-analysis were not associated with an excess mortality caused by cardiac disease or leukemia.^2^ A more prolonged follow-up may however be necessary to demonstrate anthracycline associated cardiac toxicity.^8^ A joint analysis of individual-patient data from several trials suggested that patients with tumors expressing amplification of *TOP2A*, encoding topoisomerase II alpha (a primary anthracycline target), preferentially benefit from anthracycline-based chemotherapy as compared to CMF.^9,10^ However, a suboptimal reproducibility has together with a technical-demanding and costly assay procedure prevented widespread incorporation of *TOP2A* assays into clinical practice.

The PAM50 gene set has become a standard for identifying intrinsic subtypes from RNA expression measurements, and has been developed into a distributed clinical test, the Prosigna Prognostic Gene Signature Assay, validated and health regulator-cleared to estimate the prognosis for post-menopausal breast cancer patients with ER-positive early-stage breast cancer.^11–13^ Data from National Cancer Institute of Canada Clinical Trials Group (NCIC.CTG) MA.5 trial comparing adjuvant cyclophosphamide, epirubicin and fluorouracil (CEF) versus CMF in patients with early breast cancer has suggested that the Her2-enriched subtype is associated with preferential benefit from CEF while no significant differences between treatment arms were shown for patients with Basal-like, Luminal A or Luminal B breast cancers.^14^ The purpose of the present study was to use DBCG 89D, a symmetrically designed randomized trial of CEF against CMF, to evaluate whether patients with a Her2-enriched subtype achieve a significantly better outcome with CEF compared to CMF. Analogous findings on two such similar trials would achieve a high level of evidence for clinical validity.^15^

## Results

The DBCG 89D trial enrolled 980 Danish patients, among whom 962 were treated per protocol (Supplementary Fig. 1). Tumor blocks were available from 696 patients, and 691 blocks contained sufficient amounts of invasive tumor to meet criteria for proceeding to RNA extraction. The Prosigna assay was successful in blocks from 686 patients. These 686 patients forming the study population differed significantly from the 276 non-assessable patients with regard to menopausal status (*P* = 0.01), age (*P* = 0.002), tumor size (*P* < 0.0001), grade (*P* = 0.0003), and local treatment (*P* = 0.001), although the magnitudes of these differences were small (Table 1). Median age among the 686 patients was 47 years at time of diagnosis. The histological type was mostly ductal (94%), with few lobular cancers (2%). The estimated median (interquartile range) potential follow-up was 9.8 (8.2–10.1) years for DR and 24.4 (22.4–26.8) years for OS. There were 257 DR events, 286 events for TR, and in total 392 deaths. The treatment effect favoring CEF for DR (adjusted HR 0.71, 95% CI 0.54–0.92, *P* = 0.01) was similar to the effect observed for DR in the original study population (HR 0.79, 95% CI 0.64–0.97).

**Table 1:** Patient and tumor characteristics by Prosigna (PAM50) subtype.

Characteristics	Trial population		Study population		Molecular subtype								
					Luminal A		Luminal B		Basal-like		Her2-E		*P*^a^
	No.	%	No.	%	No.	%	No.	%	No.	%	No.	%	
No. of patients	962		686		132	19	78	11	259	38	217	32	
Age													<0.0001
<40 years	163	17	97	14	17	13	13	17	46	18	21	10	
40–49 years	472	49	345	50	87	66	48	62	120	46	90	41	
50–59 years	201	21	155	23	15	11	10	13	69	27	61	28	
60–69 years	126	13	89	13	13	10	7	9	24	9	45	21	
Menopausal status													<0.0001
Pre	688	72	475	69	116	88	68	87	177	68	114	53	
Post	274	28	211	31	16	12	10	13	82	32	103	47	
Loco-regional therapy													0.02
BCS	187	19	114	17	27	20	17	22	50	19	20	9	
M −RTG	576	60	416	61	80	61	44	56	154	59	138	64	
M +RTG	197	20	154	22	25	19	17	22	53	20	59	27	
M ?RTG	2	0.2	2	0.3	0	0	0	0	2	1	0	0	
Lymph node status													<0.0001
Negative	352	37	244	36	70	53	42	54	99	38	33	15	
1–3 positive	321	33	222	32	33	25	21	27	90	35	78	36	
4+ positive	289	30	220	32	29	22	15	19	70	27	106	49	
Tumor size													0.0002
0–20 mm	422	44	272	40	75	57	32	41	94	36	71	33	
21–50 mm	462	48	357	52	52	39	41	53	145	56	119	39	
>50 mm	73	8	57	8	5	4	5	6	20	8	27	4	
Unknown	5	1	0	0	0	0	0	0	0	0	0	0	
Histologic type													0.004
Ductal	897	93	642	94	123	93	77	99	236	91	206	95	
Lobular	19	2	12	2	6	5	1	1	2	1	3	1	
Other	42	4	31	5	3	2	0	0	20	8	8	4	
Unknown	4	0.4	1	0.1	0	0	0	0	1	0.4	0	0	
Malignancy grade^b^													<0.0001
Grade I	67	7	35	5	17	14	4	5	5	2	9	4	
Grade II	474	53	335	52	89	72	59	77	73	31	114	55	
Grade III	356	40	272	42	17	14	14	18	158	67	83	40	
ER status													<0.0001
Positive	202	21	155	23	68	52	47	60	6	2	34	16	
Negative	420	44	328	48	19	14	8	10	183	71	118	54	
Unknown	340	35	203	30	45	34	23	29	70	27	65	30	
*HER2*													<0.0001
Normal	586	61	463	67	116	88	69	88	246	95	32	15	
Positive	288	30	223	33	16	12	9	12	13	5	185	85	
Unknown	88	9	0	0	0	0	0	0	0	0	0	0	
*TOP2A*													<0.0001
Deletion	87	9	67	10	8	6	3	4	12	5	44	20	
Normal	594	62	451	66	99	75	62	79	208	80	82	38	
Amplification	92	10	77	11	6	5	4	5	11	4	56	26	
Unknown	189	20	91	13	19	14	9	12	28	11	35	16	
Chemotherapy													0.18
CMF	515	54	362	53	59	45	44	56	145	56	114	53	
CEF	447	46	324	47	73	55	34	44	114	44	103	47	

*Her2-E* Her2-enriched, *BCS* breast conserving surgery, *M* mastectomy, *RT* radiotherapy, *CMF* cyclophosphamide, methotrexate and fluorouracil, *CEF* cyclophosphamide, epirubicin and fluorouracil.

^a^Test of categorical variable versus molecular subtype.

^b^Ductal carcinomas only.

### Prosigna ROR score

**Fig. 1:**
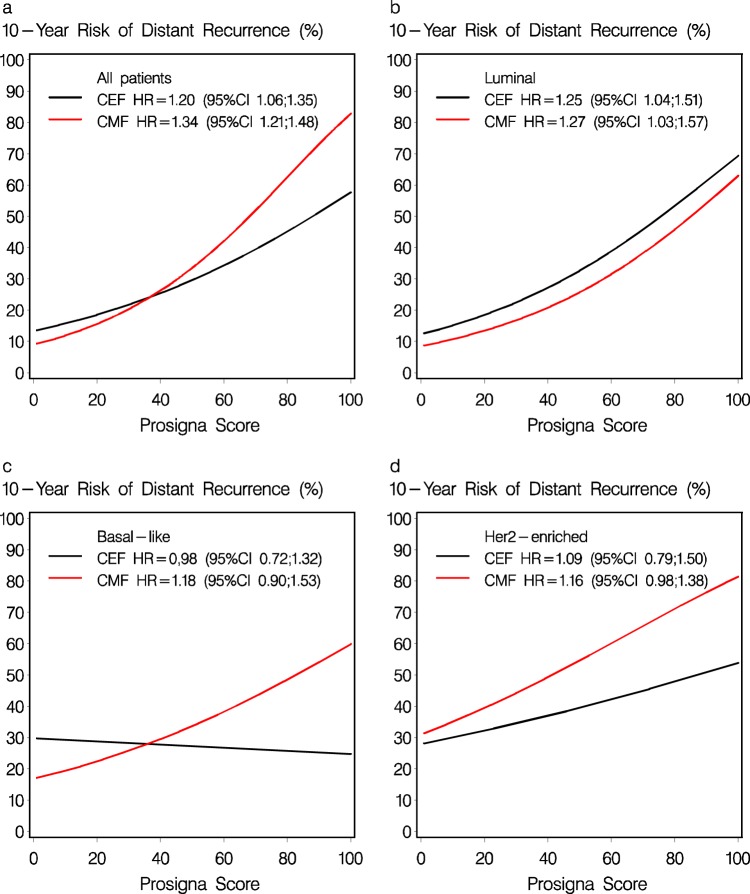
Distant recurrence rate by continuous ROR score for patients in the CMF regimen and patients in the CEF regimen. Hazard ratios and corresponding 95% CI for a 10-point difference in continuous ROR score are shown for all patients (**a**), Luminal (**b**), Basal-like (**c**) and Her2-enriched, (**d**) Prosigna breast cancer subtypes.

The relation between the continuous ROR score and 10-year DR rate is shown in Fig. 1 according to treatment regimen, with an unadjusted HR for a 10-point difference of 1.34 (95% CI 1.21–1.48, *P* < 0.0001) in CMF treated patients and HR = 1.20 (95% CI 1.06–1.35, *P* = 0.004) in the CEF treated patients (Panel a). The association of the continuous ROR score and treatment regimen in patients with Luminal subtypes is shown in Fig. 1, panel b, and the non-luminal subtypes in panels c and d. The effect of the continuous ROR score on treatment regimen was examined, and no statistically significant heterogeneity was found in unadjusted models with *P*_interaction_ = 0.16 for DR (all patients), *P*_interaction_ = 0.09 for TR and *P*_interaction_ = 0.73 for OS overall (Supplementary Fig. 2). In the multivariable models adjusting for menopausal status, tumor size, nodal status, histological type and grade, ER status and HER2 status, a significant heterogeneity was observed for TR (*P*_interaction_ = 0.02), and a borderline result for DR (*P*_interaction_ = 0.056) (Table 2).

**Table 2:** Adjusted results for ROR score continuous (10-point) according to treatment group, and unadjusted HR estimates of the treatment effect of CEF with CMF as reference according to Prosigna ROR scores and subtype.

	DR			TR			OS		
	HR	(95% CI)	*P*_interaction_	HR	(95% CI)	*P*_interaction_	HR	(95% CI)	*P*_interaction_
ROR score, continuous (10-point)			0.056			0.02			0.83
CMF	1.20	1.09;1.33		1.18	1.07;1.30		1.07	0.98;1.16	
CEF	1.04	0.92;1.18		1.00	0.89;1.13		1.06	0.96;1.16	
						<10 yrs	1.13	1.03;1.25	0.43
							1.07	0.96;1.19	
						≥10 yrs	0.92	0.79;1.07	0.41
							1.01	0.85;1.20	
Unadjusted treatment effect of CEF versus CMF									
ROR score			0.16			0.10			0.50
≤51	1.01	0.59;1.73		1.04	0.64;1.68		1.05	0.71;1.54	
52-71	0.78	0.53;1.15		0.72	0.50;1.05		0.96	0.70;1.31	
≥72	0.54	0.36;0.80		0.53	0.36;0.78		0.78	0.55;1.10	
Subtype			0.06 0.07^a^			0.01 0.07^a^			0.03 0.04^a^
Luminal A	1.61	0.77;3.35		1.70	0.90;3.23		1.62	0.97;2.71	
Luminal B	1.09	0.54;2.19		1.03	0.54;1.97		1.41	0.80;2.47	
Basal-like	0.68	0.42;1.08		0.60	0.38;0.94		0.86	0.61;1.21	
Her2-enriched	0.57	0.40;0.83		0.56	0.39;0.81		0.72	0.52;0.99	

*CMF* cyclophosphamide, methotrexate and fluorouracil, *CEF* cyclophosphamide, epirubicin and fluorouracil, *ROR* risk of recurrence, *TR* time to recurrence, *DR* distant recurrence, *OS* overall survival, *HR* hazard ratio, *95% CI* 95% confidence interval, *P*_interaction_, *P* derived from a Wald test for heterogeneity.

^a^Test of interaction Her2-Enriched vs (Luminal A, Luminal B, Basal-like).

**Fig. 2:**
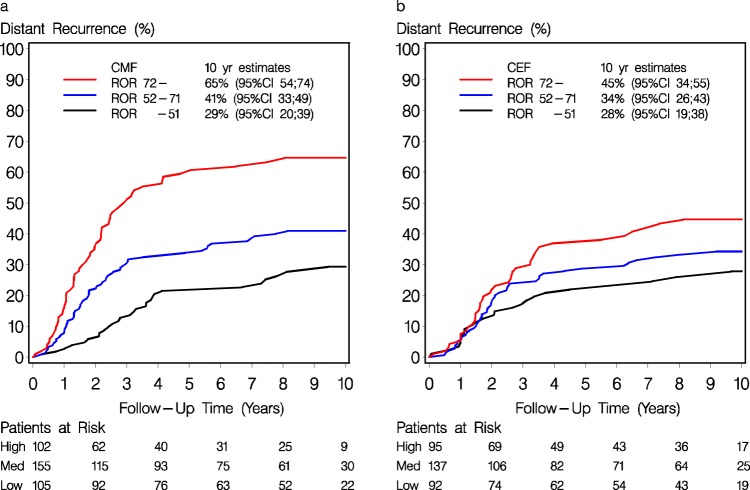
Distant recurrence rate by chemotherapy regimen and ROR score. Estimates of distant recurrence rate in patients in the CMF (**a**) and in the CEF (**b**) arm, according to Prosigna ROR ≤ 51, 52–71, and ≥72. 10-year estimates with 95% CI are included.

**Fig. 3:**
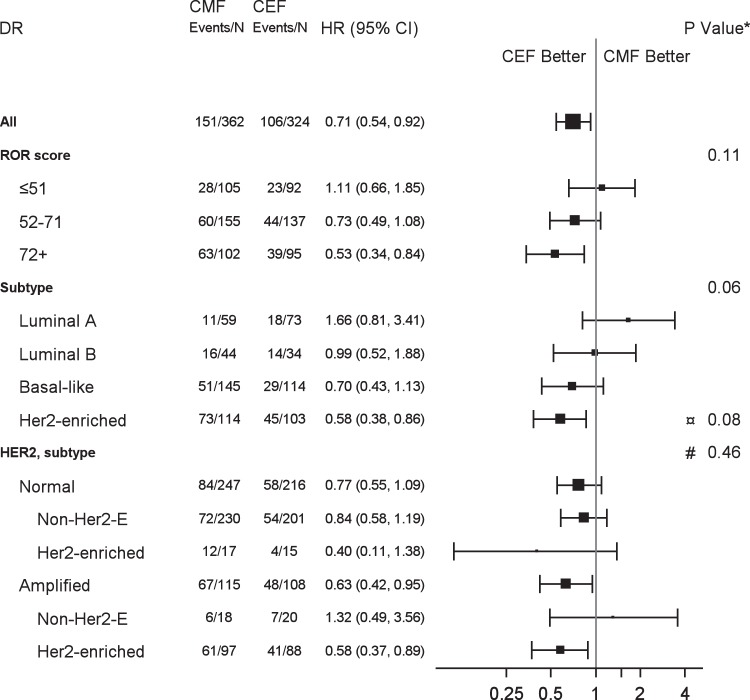
Forest plot illustrating proportional hazard models for DR according to ROR score, intrinsic subtype and HER2 status. Hazard ratios refer to adjusted estimates obtained in the multivariable analysis. Boxes represent the weight of data for each subgroup relative to the total data. *Test of interaction between treatment and subgroup (¤Her2-enriched vs rest, #HER2 Normal vs Amplified) unadjusted for multiplicity.

**Fig. 4:**
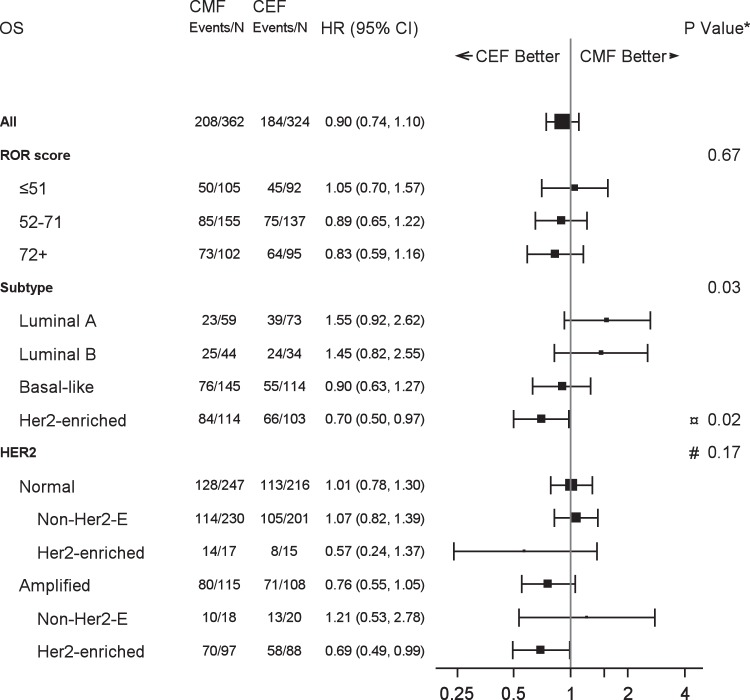
Forest plot illustrating proportional hazard models for OS according to ROR score, intrinsic subtype and HER2 status. Hazard ratios refer to adjusted estimates obtained in the multivariable analysis. Boxes represent the weight of data for each subgroup relative to the total data. *Test of interaction between treatment and subgroup (¤Her2-enriched vs rest, #HER2 Normal vs Amplified) unadjusted for multiplicity.

The pre-defined ROR score groups constituted 197 (29%) patients in the ROR ≤ 51 group, 292 (43%) patients in the ROR 52-71 group and 197 (29%) in the ROR ≥ 72 group. The DR rates are shown in Fig. 2 for the CMF and the CEF group and Table 2 shows the unadjusted results of DR, TR and OS for the treatment effect of CEF vs CMF according to ROR score group. Treatment effects were more pronounced in groups with higher ROR score, however without a statistically significant heterogeneity. The HR for treatment effect in the predefined intervals remained essentially unchanged following adjustment for patient and tumor characteristics (Figs. 3 and 4). Results for TR are shown in Supplementary Fig. 3, with a statistically significant interaction (*P*_Interaction_ = 0.045).

### Prosigna intrinsic subtype

**Fig. 5:**
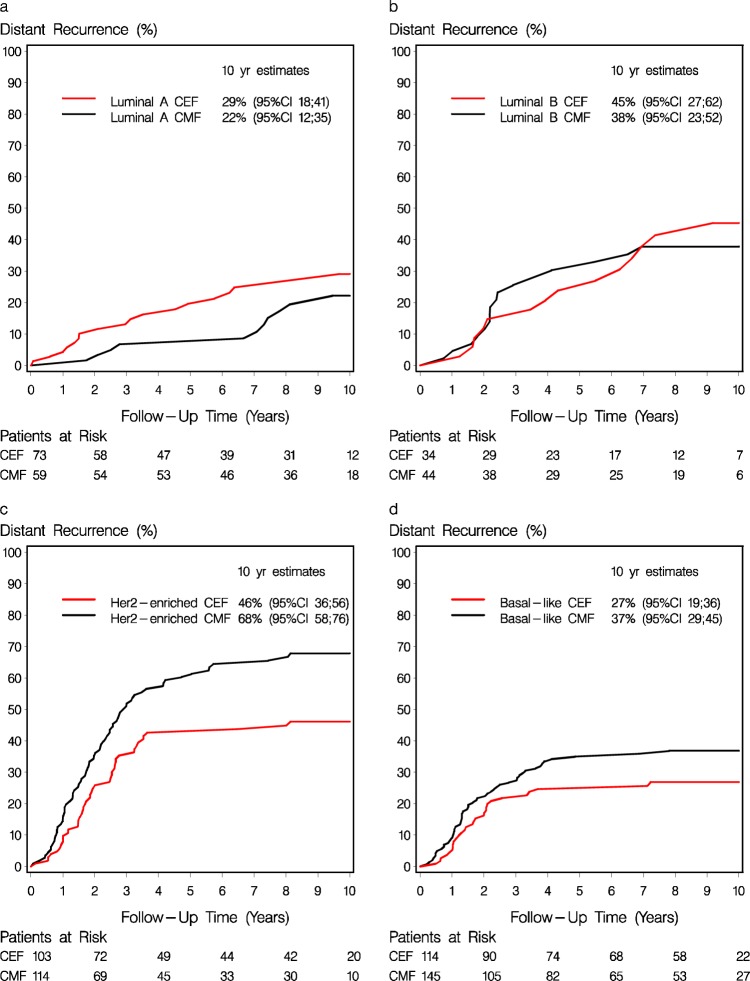
Distant recurrence rate by Prosigna subtype and treatment regimen. Estimates of distant recurrence rate according to chemotherapy regimen and intrinsic subtype for patients with Luminal A (**a**), Luminal B (**b**), Her2-enriched (**c**) and Basal-like (**d**) Prosigna breast cancer subtypes.

Table 1 shows the baseline characteristics according to Prosigna subtype. 259 (38%) were classified as Basal-like, 217 (32%) as Her2-enriched, 132 (19%) as Luminal A and 78 (11%) as Luminal B. The associations of subtype with the patient, tumor and treatment characteristics are shown in Table 1. Among the non-Her2-enriched (by gene profile) patients, 38 (8.1%) were clinically HER2 positive, and an additional 30 (6.4%) were *TOP2A* abnormal (Supplementary Table 1). Treatment outcome for DR according to Prosigna intrinsic subtype is shown in Fig. 5. Similarly, the treatment effect of CEF compared to CMF according to intrinsic subtype is shown in Table 2 for unadjusted results. The adjusted estimates in Figs. 3 and 4 show analogous results: a more favorable DR and OS was observed for patients with Her2-enriched subtype treated with CEF as compared to CMF, whereas no benefit was achieved among patients with either of the luminal subtypes. A statistically significant heterogeneity of treatment effect was observed according to PAM50 subtype for both OS (*P*_interaction_ = 0.03) as well as for TR (*P*_interaction_ = 0.02, Supplementary Fig. 3); for DR there was a comparable but not significant result (*P*_interaction_ = 0.06). Supplementary Fig. 4 shows the estimates for long-term OS for the Prosigna intrinsic subtypes and treatment. Within patients with HER2 amplified tumors only those who simultaneously had a Her2-enriched subtype appeared to benefit from epirubicin (Figs. 3 and 4), although the discordant groups were small. Within the subgroup of non-Her2-enriched breast cancers, patients with either HER2-positive or *TOP2A*-abnormal disease were limited in numbers and did not show a differential treatment effect as compared to patients with HER2 normal and *TOP2A* normal disease (data not shown).

## Discussion

Our study suggests a differential benefit from the addition of an anthracycline to CMF-based chemotherapy according to intrinsic molecular subtype as provided by Prosigna. A benefit from substituting methotrexate with epirubicin is observed among patients with Her2-enriched or Basal-like subtypes whereas there is a lack of such benefit among patients with a Luminal A or Luminal B subtypes. A compelling absolute benefit seems achievable from anthracyclines by patients with non-luminal tumors who have a high absolute risk and obtain a sizable relative reduction in distant recurrence events and mortality^2^. The Prosigna assay showed better predictive information than HER2 gene amplification. Further, ER status, differentiation, and other tumor characteristics available for the EBCTCG meta-analysis appeared to have little effect on improvement in mortality observed with anthracycline-based regimens.^1,2^ The correlation between the continuous ROR score and anthracycline benefit is consistent with greater benefit among patients with a genomic poor prognosis, and for patients with a luminal subtype the continuous ROR score showed no sign of a relationship with benefit from anthracycline chemotherapy.

While patients with a Her2-enriched subtype achieved the greatest benefit from allocation to CEF in this study, premenopausal patients with T3 or node positive breast cancer, and a Her2-enriched subtype obtained no benefit from cyclophosphamide-based chemotherapy as compared to no chemotherapy in the DBCG 77B trial.^7^ At that time, HER2 targeted therapy was not available for patients with HER2 overexpressing or amplified breast cancers, and with anti-HER2 therapy the effect of anthracyclines on the Her2-enriched subtype might be less clear. Patients with luminal breast cancer derived no significant benefit from substitution of methotrexate (in CMF) with epirubicin (in CEF), although the risk of permanent cessation of menses among premenopausal patients was higher in the CEF compared to the CMF group.^16^ Endocrine therapy was not available for patients in the DBCG 89D trial. The incremental benefit from addition of epirubicin to a taxane-based chemotherapy regimen could not be evaluated in this trial. A non-significant DR benefit of 30% from CEF compared to CMF was obtained by patients with a Basal-like subtype. This is in line with the meta-analysis from five randomized trials,^10^ where a differential benefit among patients with HER2 normal breast cancer was indicated, and the benefit of anthracycline for the triple negative subgroup seemed more pronounced compared to the results for patients with luminal subtypes. Overall our results support the findings from the NCIC-CTG MA.5 trial which identified a differential benefit from CEF compared to CMF in patients with a Her2-enriched subtype,^14^ and concurrently emphasize that anthracyclines are not an essential component of chemotherapy for patients with luminal subtypes leading to better use of an established older drug as recommended by the EBCTCG meta analysis.^1^

This study has several potential limitations. First, the retrospective evaluation of only a subset of patients introduces a risk of bias and a residual risk may persist despite adjustments in the multivariable analyses. Moderate effects may not be identified due to the limited number of patients in the subgroup analyses. Anti-HER2 treatment and taxanes were not available at the time of this study, and though one third of patients were ER-positive, endocrine therapies were not used for this patient group. Strengths of this study are the inclusion of high-risk breast cancer patients derived from a phase III randomized trial with long-term follow-up, without any prospective biomarker selection, as well as the availability of tumor tissue from more than 70% of the patients in the trial. In addition, our study adhered carefully to ReMARK guidelines^17^ and used the validated (FDA-cleared and CE-Marked) Prosigna assay following standard operating procedures as specified by the manufacturer.

In summary, we show here that the benefit of CEF as compared to CMF correlate with increasing ROR Score. More importantly, we confirm a substantial benefit from substituting CMF with CEF in patients with a Her2-enriched subtype as previously observed in the MA.5 trial. In addition, patients with a Basal-like subtype might derive benefit from an anthracycline containing regimen while a non-significant gain from CMF was obtained by patients with luminal subtypes.

## Methods

### Patients

Details of DBCG 89D trial have been published previously.^16^ In brief, this was an open-label randomized phase 3 trial. After completion of surgery, consented patients were randomized in a factorial study design to CMF, CMF plus pamidronate, CEF, or CEF plus pamidronate. Patients with completely resected unilateral invasive early-stage breast cancer were eligible if they were either (I) premenopausal patients with node-negative tumors with grade II-III malignancies, (II) premenopausal patients with node-positive and hormone receptor-negative tumors, or (III) postmenopausal patients with node-positive and hormone receptor-negative tumors. The DBCG prepared the original trial and its translational studies that have been described in detail.^18^ The Biomedical Research Ethics of the Danish Capital Region approved the original protocol as well as this supplement (V.200.1616/89 and H-15006199). For the original trial informed consent was obtained before randomization following oral and written information. The Biomedical Research Ethics of the Danish Capital Region granted dispensation from consent for this retrospective study.

### Central assessment of Prosigna

Formalin-fixed, paraffin-embedded blocks from primary excisional surgical specimens were re-collected, and NanoString-based PAM50 testing using the Prosigna methodology^11,19^ was performed to determine intrinsic subtype and ROR score. Data were extracted from the NanoString nCounter instrument by investigators blinded to patient characteristics and clinical outcome.

### Endpoints

The primary endpoint for this study was time to distant recurrence (DR). This is defined as the interval from randomization until distant recurrence, or death due to breast cancer. Death due to breast cancer without a recorded recurrence was treated as an event with the event date being the date of death. All secondary carcinomas (including contralateral breast cancer) and deaths due to causes other than breast cancer were treated as competing risk events. For local recurrences, patient records were reviewed to determine if and when there was a subsequent distant recurrence after a local recurrence. Two secondary endpoints were evaluated: time to recurrence (TR) and overall survival (OS). TR is defined as the interval from randomization until first recurrence (local or distant), or death due to breast cancer. For OS, death due to all causes are considered as an event. For OS, complete follow-up irrespective of clinical follow-up was achieved until November 1, 2018 by linkage to the Danish Central Population Registry.

### Statistical methods

The DBCG statistical office was not involved in biomarker data collection but received the biomarker data to be combined with the clinical data and executed the statistical data analysis following a written prespecified statistical analysis plan. Patient and treatment characteristics were compared according to eligibility criteria as well as biomarker data using the χ2-test, excluding unknowns. Time of follow-up was quantified in terms of a Kaplan-Meier estimate of potential follow-up.^20^ Kaplan-Meier estimates were calculated for OS and estimates of cumulative incidence for recurrence. The prognostic and predictive values were analyzed using univariable and multivariable models. Patients treated according to protocol were included. Competing**-**risk analysis using Fine and Gray’s proportional sub-distribution hazards model to evaluate the recurrence endpoints was applied. Cox proportional hazards regression modeling was used to evaluate the end-point of OS. The multivariable models included menopausal status, tumor size (continuous), nodal status (log[no of positive nodes+1]), histological type and grade, ER status (negative, positive, unknown), HER2 (normal, positive, unknown) and treatment regimen. The ROR score as a continuous measure (10-point change), categorical risk groups (ROR ≤ 51, 52–71 and ≥72) and PAM50 molecular subtypes were included in separate models. ROR cut-offs for analysis were pre-specified according to a previous study.^7^ Proportional hazards assumptions were assessed using Schoenfeld residuals and by including a time-dependent component for each covariate. The hazard rates for nodal status (OS) and for ER (TR, DR, OS) were not proportional and a time dependent component was included for nodal status, and ER was modeled for early and late periods (<5 years, ≥5 years). To comply with proportional hazards assumptions regarding subtypes and ROR score, separate estimates were included according to time since randomization: <10 years, ≥10 years for continuous ROR score and OS, <5 years, ≥5 years for subtype and TR, DR and OS. For sensitivity analysis models evaluating treatment interaction with subtype, HER2 and ER were excluded, resulting in comparable results. The Wald test was used to assess heterogeneity. *P*-values are 2-tailed, unadjusted for number of comparisons. Statistical analyses were done using the SAS 9.4 software program package (SAS Institute, Cary, NC).

### Reporting summary

Further information on experimental design is available in the Nature Research Reporting Summary linked to this article.

## Supplementary information

Supplemental material

Reporting Summary Checklist

## Data Availability

The data generated and analyzed during this study are described in the following data record: 10.6084/m9.figshare.11619069.^21^ The data supporting all the figures, tables and supplementary tables in the published article, are not publicly available due to institutional restrictions. The dataset can be made available to qualified researchers through application to the Danish Breast Cancer Cooperative Group. Please contact dbcg.rigshospitalet@regionh.dk.
