# Supplementary material for: The Prosigna 50-gene profile and responsiveness to adjuvant anthracycline-based chemotherapy in high-risk breast cancer patients
Source: NPJ Breast Cancer. 2020 Feb 26;6:7. doi: 10.1038/s41523-020-0148-0 (PMC7044229; doi:10.1038/s41523-020-0148-0)

SUPPLEMENTAL MATERIAL

Supplementary Table 1. Prosigna subtype, *TOP2A* and HER2.

	**HER2**										
	Normal					Positive					Total
	TOP2A					***TOP2A***					
	Deleted	Normal	Amplified	Unknown	Subtotal	Deleted	Normal	Amplified	Unknown	Subtotal	
**Her2-E**	1	24	3	4	32	43	58	53	31	185	217
**Non Her2-E**	**20**	350	**10**	51	431	3	19	11	5	**38**	469

Her2-E, Her2-enriched

Supplementary Figure 1. Consort Flow Diagram.

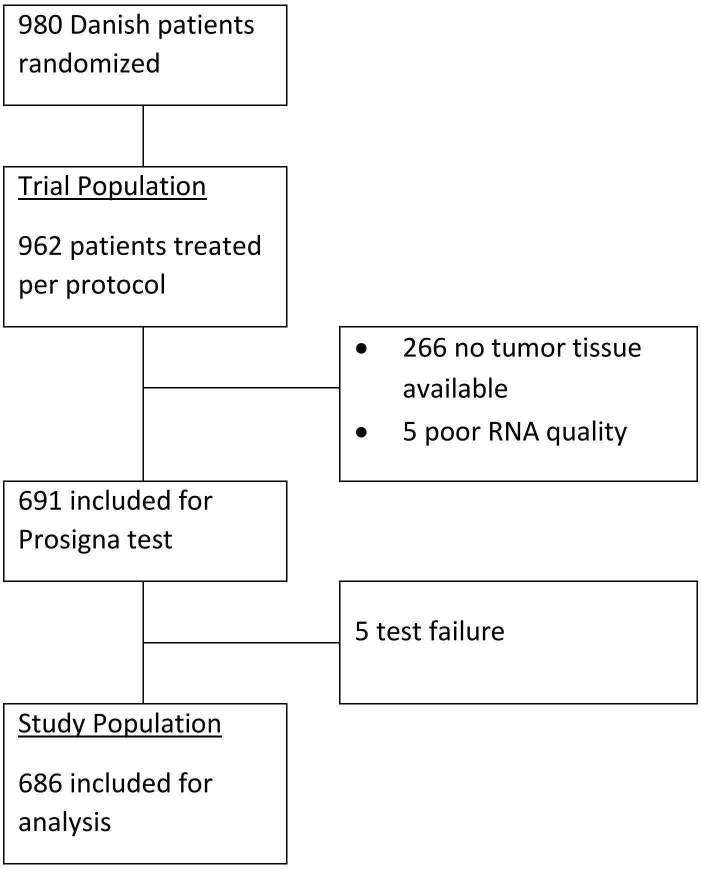

Supplementary Figure 2

Mortality rate by continuous ROR score for patients in the CMF regimen and patients in the CEF regimen. The first 10 years after inclusion (**a**), and year 10 to 25 (**b**). Hazard ratios and corresponding 95% CI for a 10-point difference in continuous ROR score are shown.

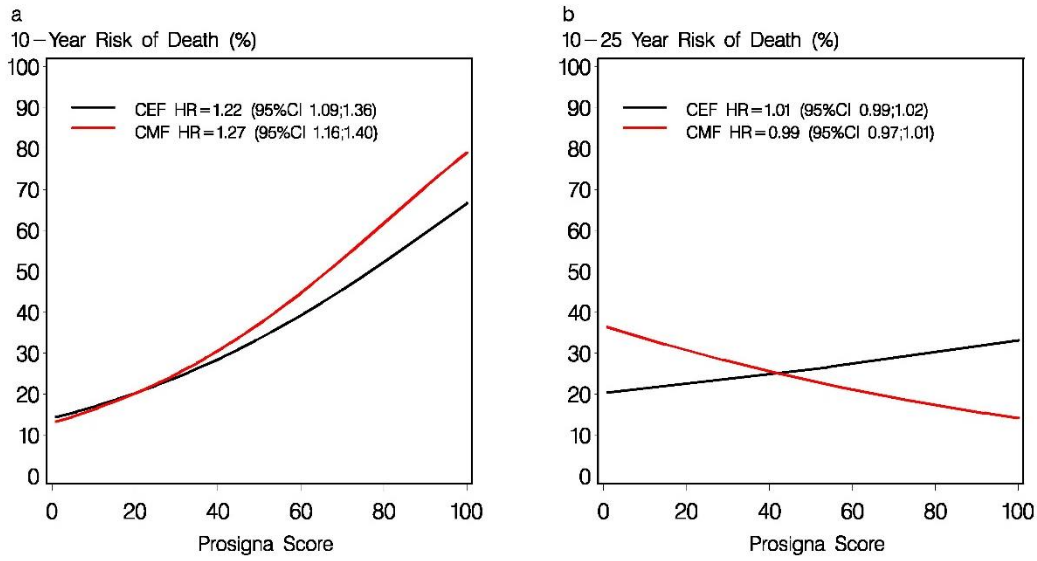

Supplementary Figure 3

Forest plot illustrating proportional hazard models for TR according to ROR score, intrinsic subtype and HER2 status. Hazard ratios refer to adjusted estimates obtained in the multivariable analysis. Boxes represent the weight of data for each subgroup relative to the total data.

TR	CMF Events/N (%)	CEF Events/N (%)	HR (95% CI)	P Value*
**All**	168/362 (46)	118/324 (36)	0.68 (0.53, 0.87)	
**ROR score**				0.04
≤51	33/105 (31)	28/92 (30)	1.12 (0.70, 1.78)	
52-71	69/155 (45)	48/137 (35)	0.67 (0.46, 0.97)	
72+	66/102 (65)	42/95 (44)	0.51 (0.33, 0.78)	
**Subtype**				0.02
Luminal A	14/59 (24)	24/73 (33)	1.70 (0.88, 3.28)	
Luminal B	19/44 (43)	16/34 (47)	0.92 (0.50, 1.70)	
Basal-like	59/145 (41)	31/114 (27)	0.62 (0.39, 0.98)	
Her2-enriched	76/114 (67)	47/103 (46)	0.53 (0.36, 0.78)	¤ 0.05
**HER2**				# 0.61
Normal	98/247 (40)	66/216 (31)	0.72 (0.52, 0.99)	
Non-Her2-E	86/230 (37)	62/201 (31)	0.77 (0.57, 1.08)	
Her2-enriched	12/17 (71)	4/15 (27)	0.33 (0.10, 1.12)	
Amplified	70/115 (61)	52/108 (48)	0.63 (0.43, 0.93)	
Non-Her2-E	6/18 (33)	9/20 (45)	1.74 (0.68, 4.43)	
Her2-enriched	64/97 (66)	43/88 (49)	0.54 (0.36, 0.83)	

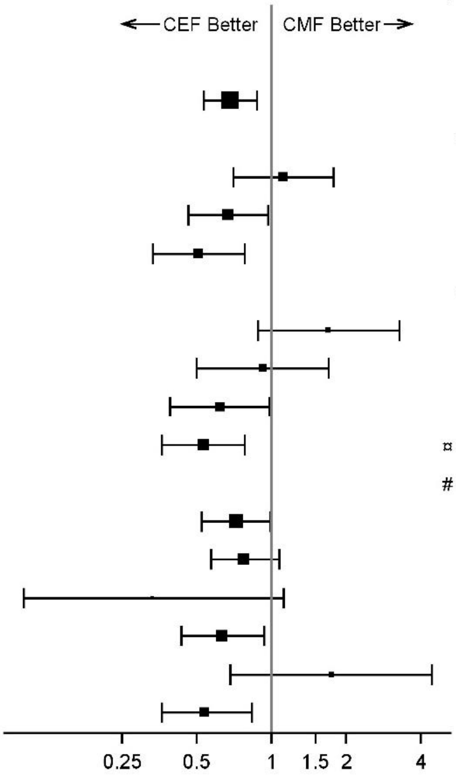

*Test of interaction between treatment and subgroup (¤Her2-enriched vs rest, #HER2 Normal vs amplified) unadjusted for multiplicity

Supplementary Figure 4

Estimates of overall survival according to chemotherapy regimen and intrinsic subtype for patients with Luminal A (**a**), Luminal B (**b**), Her2-enriched (**c**) and Basal-like (**d**) Prosigna breast cancer subtypes.

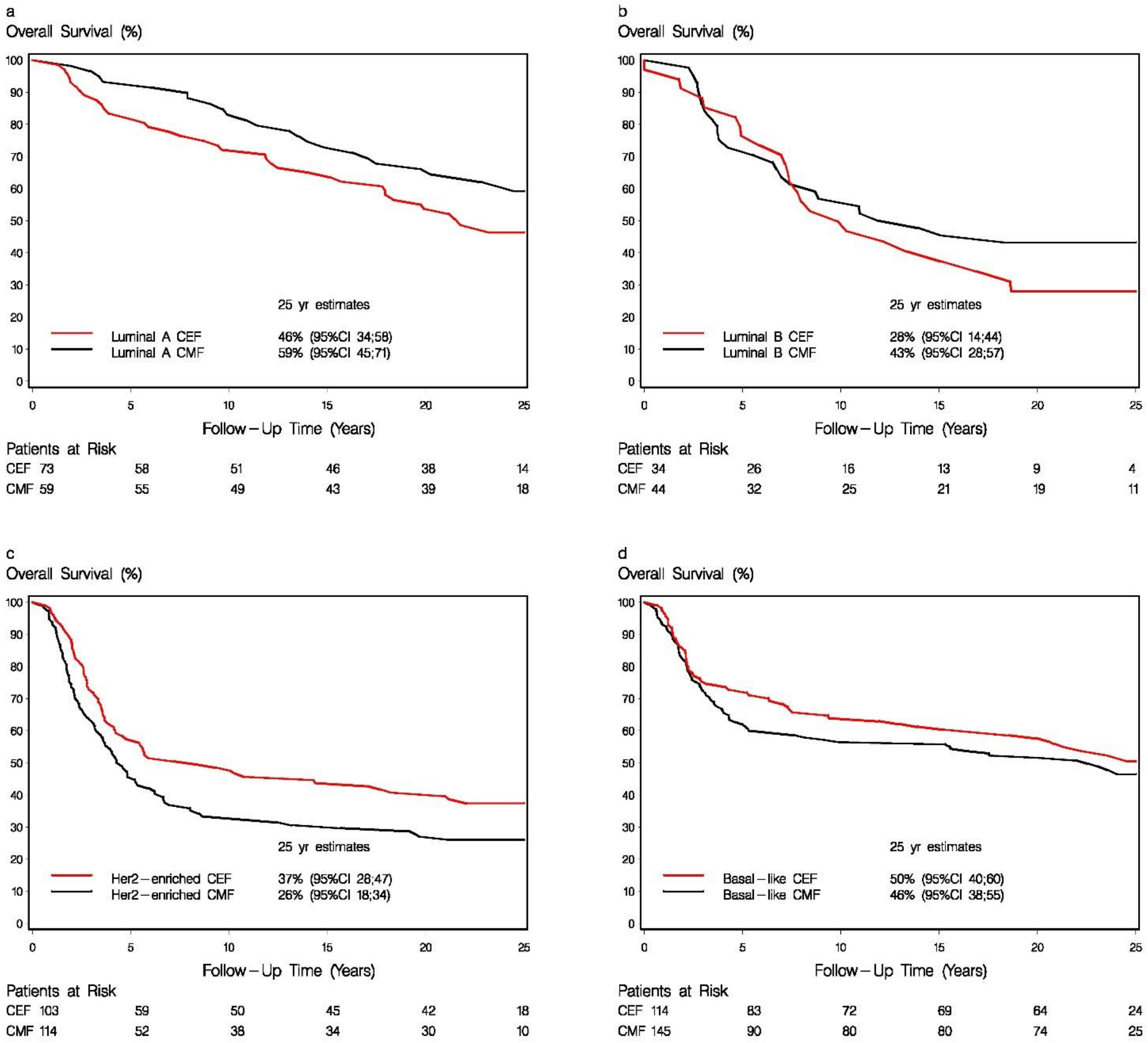

Supplement: Supplementary file 1 — Supplemental material [file 41523_2020_148_MOESM1_ESM.pdf]
